# Development and Validation of a Cognitive-Motor Assessment Using the Thinking in Motion Model

**DOI:** 10.1093/geroni/igaf122.115

**Published:** 2025-12-31

**Authors:** Shiri Embon Magal, Kfir Asraf, Maayan Agmon

**Affiliations:** University of Haifa, Haifa, HaZafon, Israel; Max Stern Yezreel Valley College, Yezreel Valley, HaZafon, Israel; University of Haifa, Haifa, HaZafon, Israel

## Abstract

The effect of cognitive-motor training on motor and cognitive functions across the lifespan is well established. However, these domains are typically assessed separately, limiting our ability to evaluate their cumulative impact. This study evaluates a novel cognitive-motor assessment: Thinking in Motion (TIM). Sixty-seven healthy adults (mean age 52.8 ± 6.1 years, age range 45–66, 67.37% women) underwent cognitive testing (Trail Making Test, Stroop Test) and motor assessments, including the burpees test, single-leg stance test, step test, Four Square Step Test (FSST), and Dual-task Tandem walking test (DT). The TIM assessment is based on a novel graphic language for movement representation, integrating various manipulations affecting execution speed, symbol interpretation, and spatial perception. As hypothesized, the TIM assessment was correlated with Stroop-A (Pearson’s r=-0.3625, p = 0.006), Stroop-B (Pearson’s r=-0.4245, p<.001), and TMT-A (Pearson’s r = 0.2951, p = 0.026), indicating its effectiveness in measuring cognitive-motor integration. Additionally, the correlation with FSST (Pearson’s r = 0.4077, p = 0.002), step test (Pearson’s r = 0.2468, p = 0.027), single-leg stance test (Pearson’s r= -0.3442, p = 0.009), DT speed (Pearson’s r=-0.2636, p = 0.05), and number of burpees (Pearson’s r=-0.2972, p = 0.025) supported its relevance in evaluating motor adaptability and postural control. TIM is a valid test that is associated with traditional measures of motor and cognitive function yet provides a broader view of cognitive-motor integration which can serve as a tool for early intervention as early as midlife. Further research should deepen the understanding of this assessment tool through long-term studies examining its predictive capabilities and its relationship with daily functional performance.

